# Comparative Epidemiology and Co-Detection of *Mycoplasma genitalium* and *Mycoplasma hominis* in Adult Urogenital PCR Testing

**DOI:** 10.3390/pathogens15090971

**Published:** 2026-09-13

**Authors:** Hyeok-Jin Kwon, Jisol Jang, Jeong Su Han, Jae-Sik Jeon, Jae Kyung Kim

**Affiliations:** 1 Department of Biomedical Laboratory Science, College of Health Sciences, Dankook University, 119 Dandae-ro, Dongnam-gu, Cheonan-si 31116, Republic of Korea; dnfgurwls@naver.com (H.-J.K.); jshan1162@naver.com (J.S.H.); zenty87@naver.com (J.-S.J.); 2 Department of Laboratory Medicine, Ewha Womans University Seoul Hospital, Seoul 07804, Republic of Korea; 3 Department of Forest Environment and Resources, College of Agriculture and Life Sciences, Chungnam National University, Daejeon 34134, Republic of Korea; shj5708@gmail.com

**Keywords:** *Mycoplasma genitalium*, *Mycoplasma hominis*, co-detection, multiplex real-time PCR, molecular epidemiology, *Gardnerella vaginalis*, diagnostic stewardship

## Abstract

Urogenital multiplex polymerase chain reaction (PCR) panels report *Mycoplasma genitalium* (MG) and *Mycoplasma hominis* (MH) similarly despite different clinical significance. We retrospectively analyzed 176,855 adult specimen-level laboratory records (2018–2022). MG and MH were compared using modified Poisson regression and a year-adjusted stacked interaction model. MG was detected in 4215 records (2.38%) and MH in 8024 (4.54%). MG prevalence fell from 4.48% (95% confidence interval (CI), 4.30–4.68) at 18–29 years to 0.21% (0.16–0.27) at ≥60 years, whereas MH fell from 5.70% to 2.39%. Female sex was associated with lower MG (adjusted prevalence ratio [aPR], 0.32) and higher MH (1.44). Age and sex gradients differed significantly (interaction PRs, 1.519 and 4.653; both *p* < 0.001), whereas no specimen-type interaction reached significance (all *p* ≥ 0.19). No other target was positive in 52.1% of MG-positive versus 54.5% of MG-negative records, but in 4.9% of MH-positive versus 55.7% of MH-negative records. MH co-detection was strongest with *Gardnerella vaginalis* (aPR, 9.94), *Ureaplasma urealyticum* (6.12), and *Trichomonas vaginalis* (5.48). These profiles support target-specific reporting. Cross-panel analytical differences cannot be excluded because MG and MH were measured on different panels.

## 1. Introduction

Multiplex real-time PCR enables simultaneous detection of multiple urogenital organisms, but a positive result does not have the same clinical meaning for every target [1,2,3,4,5,6]. This distinction is particularly important for *Mycoplasma genitalium* (MG) and *Mycoplasma hominis* (MH). MG is an established sexually transmitted pathogen associated with non-gonococcal urethritis in men and with cervicitis and pelvic inflammatory disease in women, and antimicrobial resistance complicates treatment [7,8,9,10,11]. By contrast, MH is frequently detected in asymptomatic adults and is more closely associated with bacterial vaginosis and disturbance of the vaginal microbiota; European guidance advises against routine testing or treatment of uncomplicated urogenital MH [5,12,13,14,15]. Thus, parallel presentation of MG and MH on a multiplex report should not imply equivalent epidemiology or clinical significance.

Previous Korean laboratory studies have described these organisms but have not directly quantified how their demographic and polymicrobial profiles differ. An earlier study from the same reference-laboratory archive analyzed 59,381 specimens collected from September 2018 through December 2020 and reported age-, sex-, and specimen-specific MG and MH positivity [16]. More recently, an independent national reference-laboratory analysis of 1,399,431 multiplex PCR tests performed during 2021–2024 characterized broad co-detection patterns and identified *Gardnerella vaginalis* as a central network organism [4]. Neither analysis formally tested target-by-covariate differences between MG and MH while also comparing their adjusted co-detection profiles in the same specimen-level records.

The present study analyzed 176,855 adult specimen-level laboratory records from September 2018 through December 2022 in a centralized Korean reference-laboratory dataset. A companion analysis of the same 2018–2022 adult dataset focused on *Gardnerella vaginalis* co-detection in *Ureaplasma urealyticum*-only versus *Ureaplasma parvum*-only records [17]; the present study addresses a different target pair and a different primary comparison. We asked three linked questions: whether age and sex gradients differ between MG and MH after adjustment; whether specimen type accounts for the apparent demographic contrasts; and whether MG and MH differ in the other urogenital targets detected alongside them. Because MG and MH were measured on different multiplex panels, every co-detection estimate was annotated by panel to distinguish same-reaction from cross-panel associations.

We therefore treat demographic patterns and co-detection as linked components of a single comparative question rather than as separate prevalence surveys. The dataset did not contain persistent patient identifiers, referring-institution identifiers, geographic location, or socioeconomic variables. Accordingly, the results are interpreted as specimen-level detection patterns in a reference-laboratory tested population rather than as patient-level, region-specific, or population-representative prevalence estimates.

## 2. Materials and Methods

### 2.1. Study Design and Population

We performed a retrospective analysis of urogenital multiplex real-time PCR results generated at Jangwon Medical Foundation U2Bio (Seoul, Republic of Korea), a centralized reference laboratory that receives specimens from primary and secondary medical institutions in the Republic of Korea. Records in the study dataset from September 2018 through December 2022 were eligible. The de-identified dataset did not retain referring-institution identifiers, geographic location, or socioeconomic or other social-composition variables; therefore, regional sample counts and social composition could not be characterized, and the referral setting should not be interpreted as a population-representative geographic sampling frame. Reporting followed the STROBE statement and was informed by the RECORD extension for studies using routinely collected health data [18,19]. The completed STROBE checklist is provided as Supplementary File S1.

The analysis was restricted to adults aged ≥18 years, because the question concerns adult testing practice and pediatric testing differs in referral pathways, indications, and interpretation. Of 177,599 imported records, 744 were excluded because age was <18 years, leaving 176,855 adult specimen-level laboratory records. The de-identified research dataset supplied for this analysis did not contain a reliable persistent patient identifier or a referring-institution identifier. Consequently, the investigators could not perform patient-level deduplication, link repeated submissions, or model within-patient or within-institution correlation from the analytic file; any upstream deduplication applied before data delivery could not be independently verified from the supplied dataset.

The laboratory archive has supported several prior target-specific or subgroup analyses. An earlier study analyzed 59,381 specimens collected from September 2018 through December 2020 and described MG and MH positivity by age, sex, and specimen type [16], and a separate descriptive report summarized co-infection counts among pathogen-positive specimens over 2018–2020 [20]. A 2025 Pathogens report analyzed HSV-1/HSV-2 patterns in the 2018–2022 U2Bio archive [21], and a companion study examined *G. vaginalis* co-detection in *U. urealyticum*-only versus *U. parvum*-only records from the same 2018–2022 adult dataset [17]. The earlier reports described the archive at the level of submitted specimens; because the research dataset supplied for the present analysis contained no persistent patient identifier, records are described here as specimen-level laboratory records rather than as patients. The present study extends the earlier descriptive MG/MH work to the complete 2018–2022 adult dataset and addresses a distinct primary question: formal MG-versus-MH target-by-covariate interaction testing and panel-annotated multivariable co-detection analysis. Those analyses and effect estimates have not been reported in the cited studies.

### 2.2. Laboratory Records and Analytical Variables

One analytical record represented the complete laboratory result set for one submitted urogenital specimen. DNA was extracted once per specimen and used as the common template for all three multiplex PCR panels. Results for all 12 targets were linked within each row of the study dataset, and each populated row was analyzed once. Every record carried a reported result for all 12 targets; MG and MH were therefore assessed in the same specimens and had the same denominator except for two non-evaluable MG results. Age, sex, specimen type (urine, swab, semen, or other), collection year, and the qualitative result for each target were obtained from the laboratory information system. Specimen type was supplied in the de-identified dataset as urine, swab, semen, or a residual “other” category; the component specimen labels within “other” were not retained, so this category could not be subdivided further.

Age was grouped as 18–29, 30–39, 40–49, 50–59, and ≥60 years for the main analyses, and additionally in decade bands (18–29 through ≥70 years) for age-by-sex display. Age was also modeled as a continuous variable centered at 40 years and scaled per 10 years. A result that did not meet the manufacturer-defined interpretation criteria, such as internal-control failure, was classified as non-evaluable and excluded from the denominator for the corresponding target only; non-evaluable results were rare (MG, *n* = 2; MH, *n* = 0). Co-detection was defined as simultaneous detection of two targets in the same specimen-level laboratory record.

### 2.3. Nucleic Acid Extraction

Clinical specimens were stored at −70 °C until nucleic acid extraction. For each submitted specimen, genomic DNA was extracted once using the ExiPrep™ Dx Bacteria Genomic DNA Kit (Bioneer, Daejeon, Republic of Korea) according to the manufacturer’s instructions. The extracted DNA served as the common template for the STI multiplex real-time PCR panel workflow.

### 2.4. Multiplex Real-Time PCR Analysis

The extracted DNA was tested with three four-target multiplex real-time PCR panels, all of which were performed for every record in the study dataset. The panels were the AccuPower^®^ STI8A-Plex Real-Time PCR Kit (*Chlamydia trachomatis*, *Neisseria gonorrhoeae*, *Ureaplasma urealyticum*, and MG) [22], the AccuPower^®^ STI8B-Plex Real-Time PCR Kit (*Trichomonas vaginalis*, MH, herpes simplex virus type 1, and herpes simplex virus type 2) [23], and the AccuPower^®^ STI4C-Plex Real-Time PCR Kit (*Candida albicans*, *Gardnerella vaginalis*, *Ureaplasma parvum*, and *Treponema pallidum*) [24] (all Bioneer, Daejeon, Republic of Korea). MG was therefore measured with STI8A-Plex and MH with STI8B-Plex; the two organisms compared in this study were measured on different multiplex panels performed on the same DNA extract.

Amplification and fluorescence detection were performed on an Exicycler™ 96 Real-Time Quantitative Thermal Block (Bioneer, Daejeon, Republic of Korea) according to the manufacturer’s protocol. The cycling program was a two-step protocol: initial denaturation at 95 °C for 5 min, followed by 45 cycles of denaturation at 95 °C for 5 s and combined annealing/extension at 55 °C for 5 s; no separate third elongation step was used. Results were interpreted as positive or negative using manufacturer-defined cycle-threshold criteria. The panels include positive, no-template, and internal positive controls; the internal positive control served as an amplification control rather than as an endogenous cell-adequacy marker [22,23,24]. During the study period, testing was performed as part of routine diagnostic practice within the laboratory’s established quality-assurance framework, including participation in external quality assessment and proficiency-testing programs of the College of American Pathologists and the Korean Association of External Quality Assessment Service. The research dataset contained final qualitative calls and non-evaluable flags, but not raw cycle-threshold values, run-level quality-control data, detailed target-level EQA/PT records, or historical assay- and matrix-specific validation documentation.

### 2.5. Statistical Analysis

Continuous variables were summarized as the median (interquartile range [IQR]), and categorical variables as counts with percentages. Detection prevalence and its 95% confidence intervals (CIs) were estimated using the Wilson score method. Monotone trends across ordered age groups were assessed with the Cochran–Armitage test.

Adjusted prevalence ratios (aPRs) were estimated by fitting Poisson generalized linear models with a log link and using HC0 heteroskedasticity-robust sandwich standard errors. Prevalence differences were estimated with linear probability models, and odds ratios were estimated by logistic regression; HC0 heteroskedasticity-robust sandwich standard errors were also used for these secondary effect-scale models. Adjusted models for MG and MH included age group, sex, specimen type, and collection year as categorical covariates. HC0 standard errors provide heteroskedasticity-robust inference at the laboratory-record level but do not account for correlation among repeated submissions from the same person or institution, because patient- and institution-level clustering identifiers were unavailable.

To compare MG and MH directly rather than by inspection of two separate models, each specimen-level laboratory record contributed two observations to a stacked dataset, one for MG and one for MH. A single modified Poisson model included a target indicator; continuous age, sex, specimen type, and collection-year main effects; and target-by-age, target-by-sex, target-by-swab, target-by-other, target-by-semen, and target-by-year interaction terms. For this stacked model, a cluster-robust sandwich covariance estimator was used with specimen-level laboratory record as the clustering variable, thereby accounting for the paired MG and MH observations contributed by each record. An interaction PR of 1.0 indicates identical covariate effects for the two organisms; values away from 1.0 quantify the between-target contrast.

Co-detection was quantified by fitting, for each of the ten remaining targets, a modified Poisson model for MG (and separately for MH) that included the co-detected target together with continuous age, sex, specimen type, and collection year. Each co-detected target was annotated as measured on the same multiplex panel as the index organism or on a different panel, so that associations that could reflect within-reaction effects are distinguishable from those that cannot. The 20 co-detection *p* values were adjusted together using the Benjamini–Hochberg false discovery rate (FDR) procedure. The polymicrobial context of each organism was summarized by the mean number of positive calls among the other 11 reported targets and by the proportion of index-positive records in which no other positive target was recorded. Records with a non-evaluable result for either the index target or the co-detected target were excluded from the corresponding comparison.

A sensitivity analysis repeated the principal comparisons among urine records, the specimen category with the least apparent confounding by sex-specific collection practice. All tests were two-sided; *p* < 0.05 was used for the primary comparisons and interaction tests, and FDR-adjusted *p* < 0.05 for the co-detection comparisons. Analyses were performed using R version 4.3.3 (R Foundation for Statistical Computing, Vienna, Austria). ChatGPT-5.6 (OpenAI, San Francisco, CA, USA; web application, accessed 27 August 2026) was used solely for English-language editing and refinement of the manuscript. ChatGPT was not used for study conception, generation of research ideas, statistical analysis, data processing, interpretation of results, or generation of study data. All analyses, interpretations, and reported results were independently performed, reviewed, and verified by the authors.

### 2.6. Analytical Scope

The unit of analysis was the specimen-level laboratory record because the supplied research dataset did not contain reliable patient or referring-institution identifiers; patient-level linkage, additional deduplication, and clustering by person or institution therefore could not be performed by the investigators. As a laboratory-based tested population rather than a population sample, the estimates represent detection prevalence among submitted specimens. Individual testing indications, referring-institution locations, and socioeconomic variables were not available in the research dataset. Only qualitative results were available, MG and MH were measured on different multiplex panels, and specimen type was strongly associated with sex. These constraints limit interpretation to laboratory co-detection patterns and preclude inference about patient-level or regional prevalence, assay equivalence, causality, or clinical utility.

### 2.7. Ethics Statement

The Institutional Review Board of Jangwon Medical Foundation approved the study (IRB No. 2023066; 27 December 2023) and waived informed consent for the retrospective use of de-identified laboratory records. The study was conducted in accordance with the Declaration of Helsinki.

## 3. Results

### 3.1. Study Population and Overall Detection

A total of 176,855 adult specimen-level laboratory records generated between 2018 and 2022 were analyzed (Table 1). The median age was 39.0 years (IQR, 29.0–53.0; range, 18–99); 40,348 records (22.8%) were from women and 136,507 (77.2%) from men. Specimens comprised urine (142,503; 80.6%), swabs (21,730; 12.3%), a residual “other” category (12,250; 6.9%), and semen (372; 0.2%). At least one of the 12 reported targets was detected in 82,745 records (46.79%). MG was detected in 4215 of 176,853 evaluable records (2.38%; 95% CI, 2.31–2.46) and MH in 8024 of 176,855 (4.54%; 4.44–4.64). MG and MH were co-detected in 259 records (0.15%).

### 3.2. Detection Prevalence by Age, Sex, Specimen Type, and Year

MG prevalence declined steeply across age groups, from 4.48% (95% CI, 4.30–4.68) at 18–29 years to 0.21% (0.16–0.27) at ≥60 years, a more than twenty-fold difference (Cochran–Armitage *z* = −43.4; *p* < 0.001). MH declined far more gradually over the same range, from 5.70% (5.49–5.92) to 2.39% (2.22–2.57), a 2.4-fold difference (*z* = −19.4; *p* < 0.001). The two organisms also showed opposite sex-specific detection patterns: MG prevalence was 2.80% in men versus 0.98% in women, whereas MH prevalence was 3.74% in men versus 7.23% in women (Table 2).

### 3.3. Age and Sex Patterns

**Figure 1:**
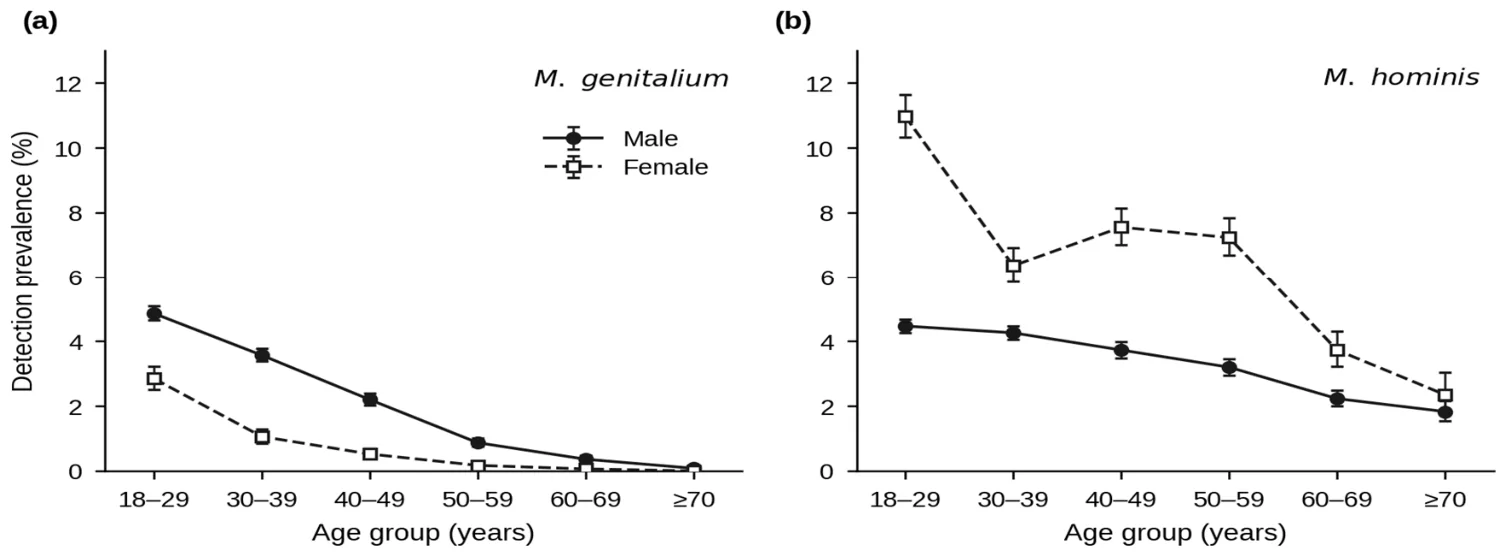
Age- and sex-specific detection prevalence of *Mycoplasma genitalium* (**a**) and *Mycoplasma hominis* (**b**). Filled circles with solid lines denote men and open squares with dashed lines women; error bars are Wilson 95% confidence intervals. Numerical values are given in Table S1.

Decade-band analysis stratified by sex showed that the two gradients diverged throughout adult life (Figure 1; Table S1). MG prevalence in men fell from 4.86% at 18–29 years to 0.08% at ≥70 years, and in women from 2.85% to 0.00% (0 of 2427 records); men had higher MG prevalence in every age band. MH prevalence in women declined overall from 10.97% to 2.35%, but the gradient was not strictly monotonic: prevalence fell to 6.36% at 30–39 years, increased to 7.55% at 40–49 years, and then declined again. In men, MH prevalence decreased steadily from 4.47% to 1.83%; women had higher MH prevalence in every age band. Neither organism showed an increase in the oldest age groups.

Because swabs were obtained predominantly from women and urine predominantly from men, specimen type was a potential explanation for the observed sex contrasts. However, stratified analyses suggested that specimen type did not fully explain these differences. Within urine records, MH prevalence was 5.05% in women and 3.78% in men, whereas MG prevalence was 2.81% in men and 0.51% in women; within swab records, MH prevalence was 9.28% in women and 3.77% in men (Table S2). The direction of both sex contrasts was preserved within every specimen stratum in which both sexes were represented.

### 3.4. Multivariable Analysis

After adjustment for age group, sex, specimen type, and collection year, MG prevalence decreased monotonically with age, reaching aPR 0.05 (95% CI, 0.04–0.07) at ≥60 years relative to 18–29 years; the corresponding MH estimate was 0.44 (0.41–0.48), roughly nine times higher (Table 3). Female sex was associated with lower MG prevalence (aPR, 0.32; 0.28–0.37) and higher MH prevalence (aPR, 1.44; 1.36–1.54). Swab specimens were associated with higher prevalence of both organisms (MG, 1.49; MH, 1.72). For MG, the adjusted estimate reversed the crude comparison (1.43% in swab versus 2.53% in urine records), largely because 96.7% of swabs were obtained from women (Table S2), among whom MG detection was lower; the positive swab association became apparent after adjustment for sex and the other covariates. Estimates on the odds and prevalence-difference scales agreed in direction for every covariate.

### 3.5. Formal Contrast Between MG and MH

The year-adjusted stacked model confirmed that the differences between the two organisms were not artifacts of separate modeling (Table 4). The age effect was PR 0.565 (95% CI, 0.550–0.580) per 10 years for MG; the corresponding MH estimate was 0.858, obtained from the target-by-age interaction of 1.519 (1.474–1.566; *p* < 0.001). Thus, under the log-linear specification, the average per-decade decrease was approximately 44% for MG and 14% for MH. These per-decade ratios summarize a log-linear age term. The categorical estimates in Table 2 show that the MG gradient steepens with advancing age rather than remaining constant, so the single per-decade ratio understates the decline beyond 50 years; the contrast with MH is correspondingly conservative. The target-by-sex interaction was 4.653 (4.005–5.405; *p* < 0.001), confirming strongly divergent and oppositely directed sex effects.

None of the specimen-type interactions were statistically significant: swab versus urine, 1.118 (0.946–1.321; *p* = 0.192); other versus urine, 0.936 (0.803–1.092; *p* = 0.401); and semen versus urine, 1.932 (0.483–7.735; *p* = 0.352). These results provide no statistical evidence that specimen effects differed between MG and MH. This matrix-focused comparison does not exclude differential analytical performance between the two assays.

Each specimen-level laboratory record contributes one MG observation and one MH observation. Main-effect terms are the covariate effects for MG; interaction terms (×MH) express the ratio of the covariate effect for MH to that for MG, so a value of 1.0 indicates identical gradients. The specimen interactions compare each non-urine specimen effect with urine between MH and MG. Continuous age was centered at 40 years; therefore, the “MH vs. MG” main effect represents the between-target contrast at age 40 years for male urine records in the reference year 2018 (September–December only). Collection-year main effects and target-by-year interaction terms were included to allow target-specific temporal adjustment. PR, prevalence ratio; CI, confidence interval.

### 3.6. Co-Detection Profiles

**Figure 2:**
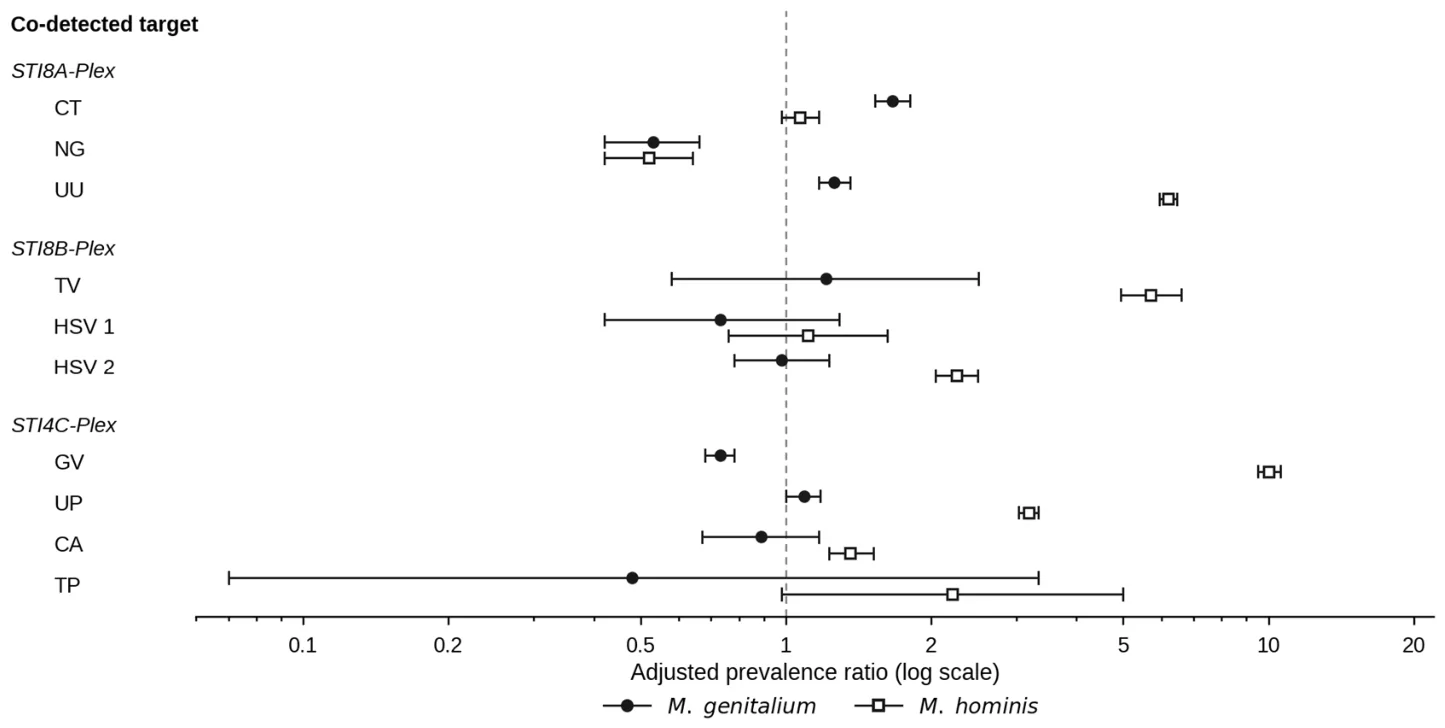
Adjusted prevalence ratios for co-detection of *Mycoplasma genitalium* and *Mycoplasma hominis* with the remaining ten reported targets, grouped by the multiplex panel on which each co-detected target was measured. STI8A-Plex contains the MG target and STI8B-Plex contains the MH target. Filled circles denote MG and open squares MH; horizontal bars are 95% confidence intervals, and the dashed vertical line marks the null value of 1.0. Numerical estimates are given in Table 5, where the target abbreviations are also defined.

MG and MH were co-detected in 259 records, corresponding to 6.1% of MG-positive and 3.2% of MH-positive records. Their co-detection profiles with the remaining ten targets, however, differed sharply (Table 5; Figure 2). MG was positively associated with *C. trachomatis* (aPR, 1.59; 95% CI, 1.46–1.73) and *U. urealyticum* (1.25; 1.16–1.35), and inversely associated with *G. vaginalis* (0.69; 0.64–0.74) and *N. gonorrhoeae* (0.53; 0.43–0.66). MH was strongly associated with *G. vaginalis* (9.94; 9.41–10.50), *U. urealyticum* (6.12; 5.87–6.39), *T. vaginalis* (5.48; 4.74–6.33), and *U. parvum* (3.18; 3.03–3.33).

The panel annotation separates associations that could arise within a single reaction from those that cannot. The three strongest MH associations other than *T. vaginalis*, namely those with *G. vaginalis*, *U. urealyticum*, and *U. parvum*, were all with targets measured on a different multiplex panel from MH, so they cannot be attributed to within-reaction effects. The inverse MG–*G. vaginalis* association was likewise cross-panel. Conversely, the two positive MG associations (*C. trachomatis* and *U. urealyticum*) were both with same-panel targets.

The polymicrobial context of the two organisms differed accordingly (Table 6). No other positive target was recorded in 52.1% of MG-positive records, close to 54.5% of MG-negative records; the mean number of other targets was 0.79 and 0.72, respectively. By contrast, no other positive target was recorded in 4.9% of MH-positive records versus 55.7% of MH-negative records; the corresponding mean numbers of other targets were 1.91 and 0.65.

For each index organism, “other targets” comprises all 11 remaining reported targets, including the other mycoplasma. Only positive calls contributed to the target count. The two non-evaluable MG records were excluded from the MG-status comparison. SD, standard deviation.

### 3.7. Temporal Pattern and Urine-Only Sensitivity Analysis

**Figure 3:**
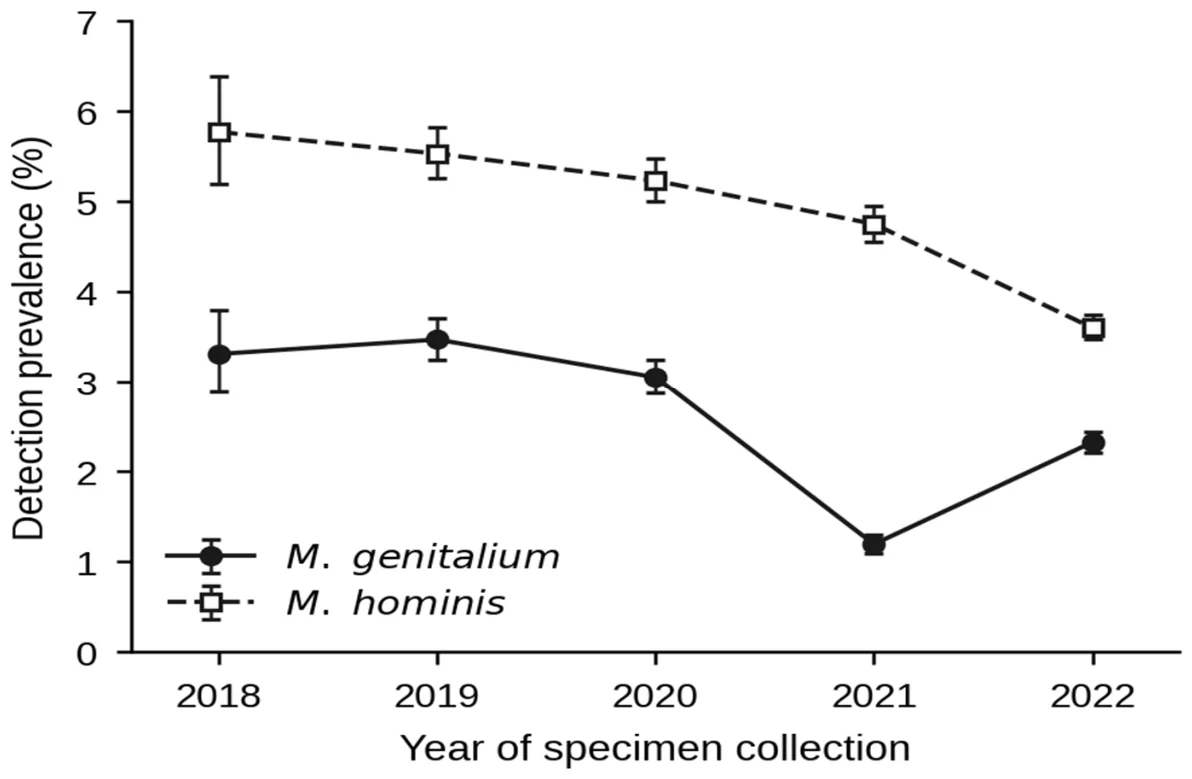
Annual detection prevalence of *Mycoplasma genitalium* and *Mycoplasma hominis*, 2018–2022. Data for 2018 cover September through December only. Error bars denote Wilson 95% confidence intervals.

MH prevalence declined across the study period, from 5.77% (95% CI, 5.20–6.39) in 2018 to 3.60% (3.47–3.74) in 2022 (aPR for 2022 versus 2018, 0.61; 0.54–0.68). MG prevalence was comparatively stable through 2020 but fell to 1.19% (1.09–1.30) in 2021 (aPR, 0.39; 0.33–0.45) before recovering in part to 2.32% (2.21–2.43) in 2022 (Figure 3). The annual patterns are presented descriptively; target-by-year terms were included in the stacked model for adjustment but are not interpreted as formal between-target temporal contrasts. The 2021 MG trough falls within the period of coronavirus disease 2019 control measures in the Republic of Korea. Reported chlamydia infections in the Republic of Korea fell by about 30% during the pandemic period, a decrease the authors considered likely to reflect underdiagnosis and underreporting [25]. The present data cannot separate these explanations, and a change in referral or care-seeking is as plausible here as a change in transmission.

Restriction to urine records (*n* = 142,503) did not alter the findings (Table S3). In the separate organism-specific models adjusted for continuous age, sex, and collection year (Panel A), the age effect remained PR 0.584 (95% CI, 0.568–0.600) per 10 years for MG and 0.848 (0.833–0.864) for MH, and female sex remained associated with lower MG (0.242; 0.197–0.299) and higher MH (1.425; 1.327–1.531) prevalence. In the year-adjusted stacked model (Panel B), the target-by-age interaction was 1.453 (1.406–1.502) and the target-by-sex interaction was 5.879 (4.731–7.307), both *p* < 0.001. Panel B included collection-year main effects and target-by-year interaction terms, matching the covariate structure of the separate models. The corresponding MH estimates derived from the main and interaction terms were 0.848 for age and 1.425 for female sex. Restricting the analysis to a single specimen matrix therefore did not remove the divergence between the two organisms.

## 4. Discussion

MG and MH behaved differently in this archive in three respects. Their modeled age gradients differed substantially; under the log-linear specification, the average per-decade decrease was approximately 44% for MG and 14% for MH. Their sex gradients ran in opposite directions, with an approximately 4.6-fold difference in the size of the sex effect. Their co-detection profiles also differed markedly: the proportion with no other positive target was similar between MG-positive and MG-negative records (52.1% vs. 54.5%) but sharply lower in MH-positive than MH-negative records (4.9% vs. 55.7%).

These patterns are consistent with what is known of the two organisms. The steep age decline and male predominance of MG, together with its co-detection with *C. trachomatis*, are the expected profile of a sexually transmitted pathogen in a care-seeking population [7,8,9,10,11]. The shallow age decline of MH, its female predominance across age groups and across specimen strata in which both sexes were represented, and above all its strong cross-panel association with *G. vaginalis*, the ureaplasmas, and *T. vaginalis* correspond to an organism whose detection tracks disturbance of the urogenital microbial community [12,13,14,15,26,27,28]. The inverse MG–*G. vaginalis* association fits the same reading, and prior studies have examined associations between vaginal microbiota composition and M. genitalium, although the reported associations have been inconsistent [29]. A *Gardnerella*-led polymicrobial biofilm model offers one plausible account of the MH pattern, in which *Gardnerella* adheres early and incorporates other organisms [26,27,28]. However, qualitative PCR results cannot distinguish low-burden detection from a biofilm-associated state [26,27,28], and *G. vaginalis* can also occur within vaginal community states observed in asymptomatic women [30]. Both organisms were also inversely associated with *N. gonorrhoeae*. Given the absence of symptoms and testing indications, this shared inverse association may reflect differences in the clinical populations represented by gonorrhea-positive and gonorrhea-negative records rather than microbial antagonism.

The near-absence of solitary MH detection in this dataset (only 4.9% of MH-positive records had no other positive target) reinforces the importance of interpreting MH within a polymicrobial context. The particularly strong MH–*T. vaginalis* association is biologically notable because *T. vaginalis* can harbor viable *M. hominis* intracellularly as an endosymbiont. The trichomonad has therefore been described as a potential “Trojan horse” for *M. hominis* transmission, and this symbiosis may modify inflammatory and pathogenic phenotypes [31,32]. This mechanism provides a plausible biological context for the observed co-detection; however, the present qualitative specimen-level PCR data cannot establish intracellular localization, directionality, or causality.

Clinical studies agree. In 1272 nonpregnant women assessed with standardized criteria, MH was strongly associated with bacterial vaginosis (adjusted odds ratio, 8.01; 95% CI, 5.99–10.71) and with its component signs, whereas the ureaplasmas were associated with no symptom or sign; in women without bacterial vaginosis, MH was not associated with symptoms either [13]. This finding is directionally consistent with the strong MH–*G. vaginalis* co-detection observed here (aPR, 9.94), although the effect measures, outcomes, and study populations are not directly comparable. The nationwide Korean survey of 1.4 million multiplex PCR tests likewise found co-infections to be predominantly commensal-driven and identified *G. vaginalis* as the hub organism of the co-detection network in every age group [4]. That analysis used a different national reference-laboratory network and covered 2021–2024, so its dataset is independent of the present U2Bio archive. An earlier study from the present laboratory analyzed 59,381 specimens collected from September 2018 through December 2020 and described MG and MH positivity by age, sex, and specimen type [16]. The present analysis extends that descriptive work to the complete 2018–2022 adult dataset and, critically, tests whether the demographic gradients of MG and MH differ statistically using target-by-covariate interaction models while characterizing adjusted co-detection profiles across the full multiplex target set.

MH was detected in 3.74% of male records, and in 3.78% of male urine records. That is consistent with evidence that bacterial vaginosis-associated organisms are recoverable from the male urethra in men with non-gonococcal urethritis, that the male genitourinary microbiome is shaped by vaginal sex, and that urogenital bacteria are exchanged between partners [33,34,35]; a randomized trial reporting reduced recurrence of bacterial vaginosis after male-partner treatment points in the same direction [36]. A second point concerns older adults. Sexually transmitted infection in this group has repeatedly been described as under-recognized, in the Republic of Korea and elsewhere [37,38,39], but neither mycoplasma increased in the oldest age bands here. Whatever case exists for attention to older adults, it does not rest on a rising detection rate with age in a tested population.

The comparison rests on two features of the data. MG and MH were measured on the same DNA extract from the same specimen-level laboratory record, so the tested individual, collection time, and specimen were matched within each record. The absence of statistically significant target-by-specimen interactions provides an internal comparison that argues against a simple specimen-matrix explanation, and the urine-only sensitivity analysis further reduces this concern. These findings do not exclude assay-related differences between STI8A-Plex and STI8B-Plex.

One limitation of the assay design must be set against this. MG was measured with STI8A-Plex and MH with STI8B-Plex, and no parallel assay was available to estimate cross-panel concordance; differences in primers, probes, target regions, and analytical sensitivity could therefore contribute to the observed contrasts, and this limitation applies to the same-panel MG associations with *C. trachomatis* and *U. urealyticum* with particular force. The panel annotation in Table 5 is intended to make this explicit, not to dismiss it. A purely constant, sex-invariant difference in assay sensitivity would not by itself explain the observed target-by-sex interaction. Nevertheless, sex-related differences in organism burden, specimen composition, anatomical sampling, referral patterns, or other pre-analytical factors could still contribute. The persistence of the interaction in the urine-only analysis reduces, but does not eliminate, this concern (interaction PR, 5.879; 95% CI, 4.731–7.307).

The practical implication concerns the laboratory report rather than the assay. When MG and MH appear in adjacent lines with identical qualitative wording, that presentation may encourage similar clinical interpretation despite their different clinical significance. A short interpretive comment on MH detection, noting its association with urogenital dysbiosis, its higher detection prevalence among tested women, and that routine testing and treatment for MH are not recommended by current European guidance [5], could convey information that this analysis quantifies. Such a comment requires no change to the assay. The present study does not show that such reporting improves clinical outcomes; that question requires patient-level studies with linked indications and outcomes [5].

Some limitations follow from the design. This was a single reference-laboratory study of submitted specimens rather than a population sample, so the estimates are detection prevalences within a tested population rather than population prevalence. The de-identified dataset did not retain referring-institution identifiers, geographic location, or socioeconomic or other social-composition variables; regional contributions could therefore not be quantified, and geographic or social representativeness cannot be assumed. Although all three panels were performed for every record included in the study dataset, the laboratory-wide number and characteristics of submissions tested with only one or two panels were unavailable; selection into the three-panel dataset therefore could not be quantified. The analytical unit was the specimen-level laboratory record, and the supplied dataset lacked persistent patient and institution identifiers, so patient-level linkage, additional deduplication, and cluster modeling could not be performed. Clinical indications, symptoms, sexual history, antimicrobial exposure, and treatment outcomes were unavailable, so reading MH as a dysbiosis marker rests on co-detection patterns rather than on clinical correlation. Each co-detection model included one co-detected target at a time; because *G. vaginalis*, *U. urealyticum*, and *U. parvum* are correlated, the resulting estimates are not mutually adjusted and should be interpreted as marginal associations.

Further limitations concern measurement. Raw cycle-threshold values were unavailable, so organism load could not be examined. Quantitative organism-load data could help determine whether the observed demographic and co-detection patterns vary according to microbial burden. The assay-reported “*G. vaginalis*” result could not be resolved across Gardnerella species and genomospecies [40,41]. The residual “other” specimen group was pre-aggregated in the research dataset, so its constituent specimen types could not be examined separately; historical matrix-specific verification records for semen and this residual category were also unavailable. Differential analytical performance in these less common matrices therefore cannot be excluded. However, urine accounted for 80.6% of all records, and the principal findings persisted in the urine-only sensitivity analysis (Table S3), reducing, although not eliminating, concern that the observed contrasts were driven by specimen-matrix differences. PCR detection does not distinguish colonization from infection, and a qualitative positive result carries no information about whether an organism is present at a burden likely to matter clinically.

Multicenter studies using patient-level identifiers, prospectively defined indications, analytically comparable measurement of MG and MH, quantitative load measures, and linked clinical and microbiome data are needed to establish whether these contrasts are reproducible and whether they carry consequences for care.

## 5. Conclusions

Across 176,855 adult urogenital specimen-level laboratory records, MG and MH showed distinct demographic and co-detection profiles, with significant target-by-age and target-by-sex interactions. MG declined steeply with age, was more frequent in men, was positively associated with *C. trachomatis*, and showed a proportion with no other positive target similar to MG-negative records. MH declined only modestly with age, was more frequent in women, almost always had at least one additional target detected, and was co-detected with *G. vaginalis*, the ureaplasmas, and *T. vaginalis*. Because the two organisms were measured on different multiplex panels, cross-panel analytical differences cannot be excluded, and these results should be read as descriptive laboratory co-detection patterns rather than as evidence of a biological interaction. The divergence is nonetheless large, internally consistent, and persisted after adjustment and restriction to urine records. These findings support consideration of target-specific interpretive reporting for MG and MH rather than uniform presentation of the two organisms.

## Figures and Tables

**Table 1:** Characteristics of the adult study population and overall detection of *Mycoplasma genitalium* and *Mycoplasma hominis*.

Characteristic	*n*	Value
Adult specimen-level laboratory records, 2018–2022	176,855	100%
Age, years (median [IQR])	176,855	39.0 (29.0–53.0)
Age range, years	176,855	18–99
Sex		
Female	40,348	22.8%
Male	136,507	77.2%
Age group, years		
18–29	45,934	26.0%
30–39	43,793	24.8%
40–49	32,072	18.1%
50–59	26,705	15.1%
≥60	28,351	16.0%
Specimen type		
Urine	142,503	80.6%
Swab	21,730	12.3%
Other (residual category)	12,250	6.9%
Semen	372	0.2%
Target detection		
MG-positive	4215/176,853	2.38% (2.31–2.46)
MG non-evaluable	2/176,855	<0.01%
MH-positive	8024/176,855	4.54% (4.44–4.64)
MH non-evaluable	0/176,855	0.00%
MG and MH co-detected	259/176,853	0.15%
Any of the 12 targets detected	82,745/176,855	46.79%

IQR, interquartile range; MG, *Mycoplasma genitalium*; MH, *Mycoplasma hominis*. In the target-detection rows, *n* is given as positive (or non-evaluable) records over evaluable records for that target; elsewhere *n* is a simple count. Percentages are calculated within the stated denominator, and 95% CIs are Wilson score intervals. The residual “Other” specimen category was already aggregated in the supplied de-identified dataset; component specimen labels were unavailable and therefore could not be listed separately.

**Table 2:** Detection prevalence of MG and MH by age group, sex, specimen type, and collection year. **Panel A.** *Mycoplasma genitalium*. **Panel B.** *Mycoplasma hominis*.

(**A**) *Mycoplasma genitalium*						
**Target**	**Stratum**	**Level**	**Records, *n***	**Positive, *n***	**Prevalence, %**	**95% CI**
MG	Age group	18–29	45,934	2059	4.48	4.30–4.68
MG	Age group	30–39	43,791	1353	3.09	2.93–3.26
MG	Age group	40–49	32,072	564	1.76	1.62–1.91
MG	Age group	50–59	26,705	179	0.67	0.58–0.78
MG	Age group	≥60	28,351	60	0.21	0.16–0.27
MG	Sex	Female	40,348	397	0.98	0.89–1.09
MG	Sex	Male	136,505	3818	2.80	2.71–2.89
MG	Specimen type	Other (residual category)	12,250	293	2.39	2.14–2.68
MG	Specimen type	Semen	372	3	0.81	0.27–2.34
MG	Specimen type	Swab	21,730	310	1.43	1.28–1.59
MG	Specimen type	Urine	142,501	3609	2.53	2.45–2.62
MG	Collection year	2018	5895	195	3.31	2.88–3.80
MG	Collection year	2019	25,276	876	3.47	3.25–3.70
MG	Collection year	2020	33,720	1029	3.05	2.87–3.24
MG	Collection year	2021	42,601	508	1.19	1.09–1.30
MG	Collection year	2022	69,361	1607	2.32	2.21–2.43
(**B**) *Mycoplasma hominis*						
**Target**	**Stratum**	**Level**	**Records, *n***	**Positive, *n***	**Prevalence, %**	**95% CI**
MH	Age group	18–29	45,934	2619	5.70	5.49–5.92
MH	Age group	30–39	43,793	2044	4.67	4.47–4.87
MH	Age group	40–49	32,072	1518	4.73	4.51–4.97
MH	Age group	50–59	26,705	1166	4.37	4.13–4.62
MH	Age group	≥60	28,351	677	2.39	2.22–2.57
MH	Sex	Female	40,348	2916	7.23	6.98–7.48
MH	Sex	Male	136,507	5108	3.74	3.64–3.84
MH	Specimen type	Other (residual category)	12,250	440	3.59	3.28–3.94
MH	Specimen type	Semen	372	6	1.61	0.74–3.47
MH	Specimen type	Swab	21,730	1976	9.09	8.72–9.48
MH	Specimen type	Urine	142,503	5602	3.93	3.83–4.03
MH	Collection year	2018	5895	340	5.77	5.20–6.39
MH	Collection year	2019	25,276	1399	5.53	5.26–5.82
MH	Collection year	2020	33,720	1765	5.23	5.00–5.48
MH	Collection year	2021	42,601	2022	4.75	4.55–4.95
MH	Collection year	2022	69,363	2498	3.60	3.47–3.74

CI, confidence interval (Wilson score method). Denominators exclude non-evaluable results for the corresponding target only. Records for 2018 cover September through December only.

**Table 3:** Multivariable models for MG and MH detection: adjusted prevalence ratios, odds ratios, and prevalence differences. **Panel A.** *Mycoplasma genitalium*. **Panel B.** *Mycoplasma hominis*.

(**A**) *Mycoplasma genitalium*					
**Target**	**Variable**	**aPR (95% CI)**	**aOR (95% CI)**	**aPD, pp (95% CI)**	***p***
MG	Age 30–39 y	0.69 (0.65–0.74)	0.68 (0.63–0.73)	−1.38 (−1.63–−1.13)	<0.001
MG	Age 40–49 y	0.42 (0.38–0.46)	0.40 (0.37–0.44)	−2.59 (−2.83–−2.36)	<0.001
MG	Age 50–59 y	0.17 (0.14–0.19)	0.16 (0.14–0.18)	−3.61 (−3.82–−3.40)	<0.001
MG	Age ≥ 60 y	0.05 (0.04–0.07)	0.05 (0.04–0.06)	−4.08 (−4.28–−3.89)	<0.001
MG	Female	0.32 (0.28–0.37)	0.31 (0.27–0.36)	−1.60 (−1.73–−1.46)	<0.001
MG	Swab	1.49 (1.27–1.74)	1.51 (1.28–1.78)	0.20 (0.00–0.40)	<0.001
MG	Semen	0.24 (0.08–0.73)	0.23 (0.07–0.72)	−2.51 (−3.43–−1.58)	0.012
MG	Other (residual category)	0.91 (0.81–1.03)	0.91 (0.80–1.03)	−0.34 (−0.63–−0.05)	0.134
MG	2019	1.01 (0.87–1.18)	1.01 (0.86–1.19)	0.04 (−0.46–0.55)	0.891
MG	2020	0.91 (0.78–1.06)	0.90 (0.77–1.06)	−0.32 (−0.82–0.17)	0.206
MG	2021	0.39 (0.33–0.45)	0.37 (0.31–0.44)	−2.06 (−2.53–−1.58)	<0.001
MG	2022	0.78 (0.67–0.90)	0.77 (0.66–0.90)	−0.81 (−1.28–−0.34)	<0.001
(**B**) *Mycoplasma hominis*					
**Target**	**Variable**	**aPR (95% CI)**	**aOR (95% CI)**	**aPD, pp (95% CI)**	***p***
MH	Age 30–39 y	0.81 (0.77–0.86)	0.80 (0.75–0.85)	−1.08 (−1.37–−0.80)	<0.001
MH	Age 40–49 y	0.78 (0.74–0.83)	0.77 (0.72–0.82)	−1.24 (−1.55–−0.92)	<0.001
MH	Age 50–59 y	0.74 (0.70–0.80)	0.73 (0.68–0.79)	−1.45 (−1.78–−1.13)	<0.001
MH	Age ≥ 60 y	0.44 (0.41–0.48)	0.43 (0.39–0.47)	−3.03 (−3.31–−2.75)	<0.001
MH	Female	1.44 (1.36–1.54)	1.47 (1.38–1.57)	1.63 (1.31–1.94)	<0.001
MH	Swab	1.72 (1.60–1.85)	1.80 (1.67–1.94)	3.83 (3.35–4.31)	<0.001
MH	Semen	0.50 (0.23–1.11)	0.50 (0.22–1.11)	−1.53 (−2.82–−0.24)	0.090
MH	Other (residual category)	0.84 (0.77–0.93)	0.84 (0.76–0.93)	−0.66 (−1.02–−0.30)	<0.001
MH	2019	0.98 (0.88–1.10)	0.98 (0.87–1.11)	−0.14 (−0.80–0.52)	0.751
MH	2020	0.82 (0.73–0.92)	0.81 (0.72–0.92)	−0.98 (−1.62–−0.33)	<0.001
MH	2021	0.77 (0.69–0.87)	0.76 (0.67–0.86)	−1.26 (−1.89–−0.62)	<0.001
MH	2022	0.61 (0.54–0.68)	0.59 (0.52–0.66)	−2.28 (−2.89–−1.66)	<0.001

aPR, adjusted prevalence ratio (modified Poisson regression, log link); aOR, adjusted odds ratio (logistic regression); aPD, adjusted prevalence difference in percentage points (linear probability model). All models were mutually adjusted for age group, sex, specimen type, and collection year, with HC0 heteroskedasticity-robust standard errors. Reference categories: age 18–29 years, male sex, urine specimen, collection year 2018 (September–December only). The *p* value shown is from the modified Poisson model.

**Table 4:** Formal contrast of MG and MH in a stacked modified Poisson model with target-by-age, target-by-sex, target-by-specimen, and target-by-year interactions and specimen-level laboratory record–clustered standard errors.

Term	PR (95% CI)	*p*
Age, per 10 years (MG)	0.565 (0.550–0.580)	<0.001
MH vs. MG	1.667 (1.398–1.988)	<0.001
Age × MH	1.519 (1.474–1.566)	<0.001
Female (MG)	0.314 (0.273–0.361)	<0.001
Female × MH	4.653 (4.005–5.405)	<0.001
Swab vs. urine (MG)	1.538 (1.317–1.796)	<0.001
Swab × MH	1.118 (0.946–1.321)	0.192
Semen vs. urine (MG)	0.254 (0.082–0.784)	0.017
Semen × MH	1.932 (0.483–7.735)	0.352
Other vs. urine (MG)	0.906 (0.804–1.021)	0.104
Other × MH	0.936 (0.803–1.092)	0.401

**Table 5:** Co-detection of MG and MH with the remaining ten reported targets, annotated by multiplex panel.

Target	Co-Target	Panel	Both +, *n*	% When Co-Target +	% When Co-Target −	aPR (95% CI)	FDR *p*
MG	CT	same (STI8A)	601	6.21	2.16	1.59 (1.46–1.73)	<0.001
MG	NG	same (STI8A)	80	2.14	2.39	0.53 (0.43–0.66)	<0.001
MG	UU	same (STI8A)	774	3.33	2.24	1.25 (1.16–1.35)	<0.001
MG	TV	different (STI8B)	7	1.89	2.38	1.15 (0.55–2.39)	0.707
MG	HSV 1	different (STI8B)	12	2.68	2.38	0.71 (0.41–1.25)	0.369
MG	HSV 2	different (STI8B)	73	2.58	2.38	0.94 (0.75–1.19)	0.655
MG	GV	different (STI4C)	881	1.79	2.61	0.69 (0.64–0.74)	<0.001
MG	UP	different (STI4C)	607	2.21	2.41	1.03 (0.95–1.12)	0.556
MG	CA	different (STI4C)	51	1.51	2.40	0.91 (0.69–1.20)	0.600
MG	TP	different (STI4C)	1	2.22	2.38	0.47 (0.07–3.23)	0.556
MH	CT	different (STI8A)	490	5.06	4.51	1.04 (0.95–1.14)	0.537
MH	NG	different (STI8A)	88	2.35	4.58	0.51 (0.42–0.63)	<0.001
MH	UU	different (STI8A)	3948	16.99	2.64	6.12 (5.87–6.39)	<0.001
MH	TV	same (STI8B)	117	31.62	4.48	5.48 (4.74–6.33)	<0.001
MH	HSV 1	same (STI8B)	25	5.58	4.53	1.11 (0.76–1.61)	0.655
MH	HSV 2	same (STI8B)	331	11.68	4.42	2.20 (1.99–2.44)	<0.001
MH	GV	different (STI4C)	6428	13.07	1.24	9.94 (9.41–10.50)	<0.001
MH	UP	different (STI4C)	3263	11.87	3.18	3.18 (3.03–3.33)	<0.001
MH	CA	different (STI4C)	342	10.12	4.43	1.22 (1.09–1.35)	<0.001
MH	TP	different (STI4C)	5	11.11	4.54	2.13 (0.94–4.81)	0.116

Adjusted for age (continuous, per 10 years), sex, specimen type, and collection year, with HC0 robust standard errors. “Panel” indicates whether the co-detected target was measured on the same multiplex panel as the index organism (MG, STI8A-Plex; MH, STI8B-Plex) or on a different panel. CT, *Chlamydia trachomatis*; NG, *Neisseria gonorrhoeae*; UU, *Ureaplasma urealyticum*; TV, *Trichomonas vaginalis*; HSV, herpes simplex virus; GV, *Gardnerella vaginalis*; UP, *Ureaplasma parvum*; CA, *Candida albicans*; TP, *Treponema pallidum*; FDR, Benjamini–Hochberg false discovery rate across the 20 comparisons. Records with a non-evaluable result for either the index target or the co-detected target were excluded from the corresponding comparison. Each co-detection model included a single co-detected target; because *Gardnerella vaginalis*, *Ureaplasma urealyticum*, and *U. parvum* are themselves strongly correlated, the individual estimates are not mutually adjusted and should be read as marginal associations.

**Table 6:** Polymicrobial context by MG and MH status in specimen-level laboratory records.

Group	Records, *n*	Mean No. of Other Targets (SD)	No Other Positive Target Recorded, *n* (%)
MG-positive	4215	0.79 (1.03)	2195 (52.1%)
MG-negative	172,638	0.72 (0.95)	94,108 (54.5%)
MH-positive	8024	1.91 (0.87)	390 (4.9%)
MH-negative	168,831	0.65 (0.84)	94,110 (55.7%)

## Data Availability

The data analyzed in this study were derived from de-identified reference laboratory records maintained by U2Bio. The raw data are not publicly available because of institutional confidentiality and data-protection requirements. Aggregated de-identified data may be available from the corresponding author upon reasonable request and subject to institutional and ethical approval.

## Supplementary Materials

The following supporting information can be downloaded at: https://www.mdpi.com/article/10.3390/pathogens15090971/s1. Table S1: Age- and sex-specific detection prevalence of MG and MH in decade bands; Table S2: Detection prevalence of MG and MH by specimen type and sex; Table S3: Urine-only sensitivity analysis; File S1: Completed STROBE checklist.

## Abbreviations

The following abbreviations are used in this manuscript:

aOR	adjusted odds ratio
aPD	adjusted prevalence difference
aPR	adjusted prevalence ratio
BV	bacterial vaginosis
CI	confidence interval
FDR	false discovery rate
GV	*Gardnerella vaginalis*
IQR	interquartile range
MG	*Mycoplasma genitalium*
MH	*Mycoplasma hominis*
PCR	polymerase chain reaction
PR	prevalence ratio
SD	standard deviation
STI	sexually transmitted infection
UP	*Ureaplasma parvum*
UU	*Ureaplasma urealyticum*
