# Supplementary material for: Comparative Epidemiology and Co-Detection of Mycoplasma genitalium and Mycoplasma hominis in Adult Urogenital PCR Testing
Source: Pathogens. 2026 Sep 13;15(9):971. doi: 10.3390/pathogens15090971 (PMC13610398; doi:10.3390/pathogens15090971)
Supplement: Supplementary file 1 [file pathogens-15-00971-s001.zip › pathogens-4561375_Supplementary.pdf]

**Supplementary Materials**

Comparative Epidemiology and Co-Detection of *Mycoplasma genitalium* and *Mycoplasma hominis* in Adult Urogenital PCR Testing

Hyeok-Jin Kwon, Jisol Jang, Jeong Su Han, Jae-Sik Jeon and Jae Kyung Kim

Abbreviations: aPR, adjusted prevalence ratio; CI, confidence interval; MG, *Mycoplasma genitalium*; MH, *Mycoplasma hominis*; PR, prevalence ratio.

**Table S1. Age- and sex-specific detection prevalence of MG and MH in decade bands.**

| Target | Sex | Age group | Records, n | Positive, n | Prevalence, % | 95% CI |
|---|---|---|---|---|---|---|
| MG | Male | 18–29 | 37,232 | 1,811 | 4.86 | 4.65–5.09 |
| MG | Male | 30–39 | 35,384 | 1,264 | 3.57 | 3.38–3.77 |
| MG | Male | 40–49 | 23,653 | 520 | 2.20 | 2.02–2.39 |
| MG | Male | 50–59 | 18,976 | 166 | 0.87 | 0.75–1.02 |
| MG | Male | 60–69 | 14,010 | 51 | 0.36 | 0.28–0.48 |
| MG | Male | ≥70 | 7,250 | 6 | 0.08 | 0.04–0.18 |
| MG | Female | 18–29 | 8,702 | 248 | 2.85 | 2.52–3.22 |
| MG | Female | 30–39 | 8,407 | 89 | 1.06 | 0.86–1.30 |
| MG | Female | 40–49 | 8,419 | 44 | 0.52 | 0.39–0.70 |
| MG | Female | 50–59 | 7,729 | 13 | 0.17 | 0.10–0.29 |
| MG | Female | 60–69 | 4,664 | 3 | 0.06 | 0.02–0.19 |
| MG | Female | ≥70 | 2,427 | 0 | 0.00 | 0.00–0.16 |
| MH | Male | 18–29 | 37,232 | 1,664 | 4.47 | 4.26–4.68 |
| MH | Male | 30–39 | 35,386 | 1,509 | 4.26 | 4.06–4.48 |
| MH | Male | 40–49 | 23,653 | 882 | 3.73 | 3.49–3.98 |
| MH | Male | 50–59 | 18,976 | 607 | 3.20 | 2.96–3.46 |
| MH | Male | 60–69 | 14,010 | 313 | 2.23 | 2.00–2.49 |
| MH | Male | ≥70 | 7,250 | 133 | 1.83 | 1.55–2.17 |
| MH | Female | 18–29 | 8,702 | 955 | 10.97 | 10.33–11.65 |
| MH | Female | 30–39 | 8,407 | 535 | 6.36 | 5.86–6.91 |
| MH | Female | 40–49 | 8,419 | 636 | 7.55 | 7.01–8.14 |
| MH | Female | 50–59 | 7,729 | 559 | 7.23 | 6.68–7.83 |
| MH | Female | 60–69 | 4,664 | 174 | 3.73 | 3.22–4.31 |
| MH | Female | ≥70 | 2,427 | 57 | 2.35 | 1.82–3.03 |

CI, confidence interval (Wilson score method). These values underlie Figure 1. The age bands differ from those in Table 2: the main analyses used five groups with a combined ≥60-year category, which is split here into 60-69 and ≥70 years. Denominators exclude non-evaluable results for the corresponding target only.

**Table S2. Detection prevalence of MG and MH by specimen type and sex.**

| Target | Specimen type | Sex | Records, n | Positive, n | Prevalence, % | 95% CI |
|---|---|---|---|---|---|---|
| MG | Urine | Female | 17,300 | 89 | 0.51 | 0.42–0.63 |
| MG | Urine | Male | 125,201 | 3,520 | 2.81 | 2.72–2.90 |
| MG | Swab | Female | 21,013 | 287 | 1.37 | 1.22–1.53 |
| MG | Swab | Male | 717 | 23 | 3.21 | 2.15–4.77 |
| MG | Other | Female | 2,035 | 21 | 1.03 | 0.68–1.57 |
| MG | Other | Male | 10,215 | 272 | 2.66 | 2.37–2.99 |

| Target | Specimen type | Sex | Records, n | Positive, n | Prevalence, % | 95% CI |
|---|---|---|---|---|---|---|
| MG | Semen | Male | 372 | 3 | 0.81 | 0.27–2.34 |
| MH | Urine | Female | 17,300 | 873 | 5.05 | 4.73–5.38 |
| MH | Urine | Male | 125,203 | 4,729 | 3.78 | 3.67–3.88 |
| MH | Swab | Female | 21,013 | 1,949 | 9.28 | 8.89–9.67 |
| MH | Swab | Male | 717 | 27 | 3.77 | 2.60–5.42 |
| MH | Other | Female | 2,035 | 94 | 4.62 | 3.79–5.62 |
| MH | Other | Male | 10,215 | 346 | 3.39 | 3.05–3.76 |
| MH | Semen | Male | 372 | 6 | 1.61 | 0.74–3.47 |

Rows are ordered as in Table 1 (urine, swab, other, semen). Strata containing fewer than 50 specimen-level laboratory records are not shown. Semen specimens were obtained from men only, so no female stratum exists for that category. The direction of the sex contrast is preserved for both organisms within every specimen stratum in which both sexes are represented. The "Other" category was supplied as a pre-aggregated residual category in the de-identified research dataset; its component specimen labels were not retained and therefore could not be listed separately.

**Table S3. Urine-only sensitivity analysis.**

Panel A. Separate models for each organism, fitted within urine records.

| Target | Records, n | Positive, n | Age aPR per 10 y (95% CI) | Female aPR (95% CI) |
|---|---|---|---|---|
| MG | 142,501 | 3,609 | 0.584 (0.568–0.600) | 0.242 (0.197–0.299) |
| MH | 142,503 | 5,602 | 0.848 (0.833–0.864) | 1.425 (1.327–1.531) |

Panel B. Target-by-covariate interaction terms from a stacked model fitted within urine records.

| Interaction | PR (95% CI) |
|---|---|
| Age × MH | 1.453 (1.406–1.502) |
| Female × MH | 5.879 (4.731–7.307) |

Panel A models were adjusted for continuous age (per 10 years), sex, and collection year, with HC0 robust standard errors. Records for collection year 2018 cover September through December only. Panel B was a stacked model containing a target indicator; continuous age, sex, and collection-year main effects; and target-by-age, target-by-sex, and target-by-year interaction terms, with standard errors clustered by specimen-level laboratory record. With the matched specifications, the Panel B interaction estimates directly represent the ratios of the organism-specific effects. The corresponding MH estimates derived from the main and interaction terms were 0.848 for age and 1.425 for female sex. Both interaction terms had $p < 0.001$. The corresponding whole-cohort interaction PRs were 1.519 for age and 4.653 for sex (Table 4).
